# Comparison of Demographics: National Amyotrophic Lateral Sclerosis Registry and Clinical Trials Data

**DOI:** 10.1007/s40615-024-02047-4

**Published:** 2024-07-08

**Authors:** Moon Han, Jaime Raymond, Theodore C. Larson, Paul Mehta, D. Kevin Horton

**Affiliations:** Office of Innovation and Analytics, Agency for Toxic Substances and Disease Registry/Centers for Disease Control and Prevention, 4770 Buford Hwy NE, Atlanta, GA 30341 USA

**Keywords:** Amyotrophic lateral sclerosis, National ALS Registry, PRO-ACT, Clinical trials, Demography, Disparity

## Abstract

**Objective:**

To characterize the participant demographics in the Pooled Resource Open-Access ALS Clinical Trials (PRO-ACT) database compared with the web-portal National Amyotrophic Lateral Sclerosis (ALS) Registry (the Registry).

**Methods:**

Demographics and ALS symptom information were compared between the self-reported registrant data in the Registry web portal (2010–2021) and the latest available PRO-ACT data (updated August 2022), which is a collection of clinical trials data.

**Results:**

Greater percentages of younger (≤ 59 years old) but smaller percentages of older (60 + years old) participants were represented in PRO-ACT compared to Registry. Enrollment for minority race groups was greater in the Registry portal data, but race information was largely missing/unknown in PRO-ACT database. Median age at the time of diagnosis and age at the time of symptom onset were significantly higher for Registry enrollees compared to the participants of PRO-ACT. Symptom onset sites were similarly reported, but duration between self-noted symptom onset and diagnosis was slight, but significantly longer for the Registry enrollees (11 vs. 9 months). Hispanic were as likely as non-Hispanic to participate in research studies, based on the Registry data.

**Conclusion:**

There was a notable difference in the age distribution and minority representation of enrollees between the PRO-ACT and Registry study populations. Age distribution in the PRO-ACT database skewed to a younger and less diverse cohort. Despite the clinical heterogeneity and complex disease mechanism of ALS, identifying the underrepresented demographic niche in the PRO-ACT and Registry study populations can help improve patient participation and criteria for patient selection to enhance generalizability.

**Supplementary Information:**

The online version contains supplementary material available at 10.1007/s40615-024-02047-4.

## Introduction

Amyotrophic lateral sclerosis (ALS; Lou Gehrig disease) is a rare, progressive neuromuscular disorder that typically results in death within 2–5 years post-diagnosis, usually due to respiratory failure, with some patients surviving more than 5–10 years [1, 2]. Symptom onset due to degenerating upper and lower motor neurons leads to paralysis of voluntary skeletal muscles of limbs, face, and neck [3]. While there is no cure for ALS, a great stride in therapeutic discovery has been made since the initial Food and Drug Administration (FDA) approval of riluzole (Rilutek®) in 1995 [4]. In recent years, edaravone (Radicava®), Relyvrio™ [5, 6], and Qalsody™ (Tofersen) [7] have been approved for treating ALS in 2017, 2022, and 2023, respectively.

In 2008, the ALS Registry Act, Public Law 110–73, was mandated by Congress to better understand the disease burden among the United States (U.S) adult population [8]. In 2010, the National ALS Registry (Registry) launched an online portal as a platform for persons living with ALS to self-enroll and provide information by completing various surveys. Since then, ALS prevalence [9–15], incidence [16], and mortality [17] rates have been reported to better observe disease burden and trend by utilizing web portal and administrative national databases [e.g., Centers for Medicare and Medicaid Services (CMS), Veterans Health Administration (VHA), Veterans Benefits Administration (VBA)]. Furthermore, the online platform has been used by ALS researchers seeking to increase patient sample size and recruit for clinical trials and epidemiological studies [18, 19]. A recent study on the utilization of the Registry Research Notification Mechanism reported greater patient recruitment and enrollment compared to other methods (i.e., pamphlets and social media) [18]. However, many factors about rare disease, such as challenges of meeting the gold standard of traditional randomized clinical trials and eligibility criteria of patient recruitment, persist [20, 21]. A recent meta-analysis found 59.8% of ALS patients on average are excluded from clinical trial eligibility at the time of ALS diagnosis [22]. While reasons for such exclusion varied, the attributes of those selected for participation skewed toward more men, younger cohort, slower disease progression, and those with longer survival [22].

PRO-ACT, or Pooled Resource Open-access ALS Clinical Trials [23], represents the largest collection of publicly available Phase II/III ALS clinical trial participant data [24]. This database contains fully de-identified clinical and laboratory information collected and donated by various pharmaceutical companies and other consortia. While PRO-ACT does not represent all ALS clinical trials exclusively, an analysis of its data may provide insights into trial participant characteristics and allow examination of demographics and clinical characteristics collected in ALS clinical trials.

Our primary objective is to identify potential differences in the demographic characteristics of ALS patients being recruited through the clinical trials in PRO-ACT compared to those more likely to represent the epidemiological population as recognized in the Registry portal database. ALS clinical trial designs may be challenged by disease rarity, phenotype heterogeneity, and complex disease mechanism [25–28]. Nonetheless, learning from the barriers in clinical trials of other diseases [29–32], such as demographic disparity, and recognizing the broad spectrum of ALS population would improve the generalizability of the study outcome and approved therapeutics.

## Methods

### Data Source

Data from the Registry consists of information only from the online web-portal platform (https://www.cdc.gov/als/ALSJoinALSRegistry.html) collected from 2010 to 2021; no ALS patients’ information from CMS, VHA, or VBA was used. All responses from the Registry portal are self-reported. Demographics and ALS symptom-related information were selected for comparison with the data in the PRO-ACT.

The PRO-ACT database (https://ncri1.partners.org/ProACT/Home/Index) consists of fully de-identified ALS patient data collected since 1990 from clinical trials donated by PRO-ACT consortia (latest data update on August 2022) [23]. Data came from available select datasets: Demographics, ALSFRS-R (ALS Functional Rating Scale-Revised), Subject ALS History, Family History, and riluzole use. The PRO-ACT database design and features have been previously described elsewhere [24].

### Study Variables and Data Analysis

Databases were evaluated separately to identify variables that allowed for similar comparison. Age of participants at the time of enrollment in the Registry or the clinical trial studies conducted by PRO-ACT consortia, sex, ethnicity, and race* were selected for demographic comparisons (*See Supplemental Methods). The frequency of missing or unknown race was estimated separately to capture missing race information. ALS symptom-related variables such as age at diagnosis, age at symptom onset, site of symptom onset*, symptom duration, family history of ALS*, ALSFRS-R, and riluzole use were selected (*See Supplemental Methods). If participants had multiple ALSFRS-R entries, only the first record or an entry closer to the time of enrollment was chosen. We also extracted self-reported data of the Registry enrollees with history of ever participating in any ALS research studies. Data distribution was tested for normality, and parametric or nonparametric test was conducted accordingly. Frequency, median, percentages, and 95% confidence intervals (CIs) were calculated for descriptive statistics; Wilcoxon rank-sum or Pearson *χ*^2^ was used accordingly to test the difference in sample distribution and proportions. All analyses were performed using Stata 17 (StataCorp LLC, Texas, U.S.A).

## Results

Due to the nature of self-reporting in the Registry and consolidated clinical records collected under different trial study protocols for the data in PRO-ACT, the frequency of responses varied for each evaluation. The total number of subjects in the database was 10,471 for the Registry and 11,675 for the data in PRO-ACT (Table 1). Median age of participants in the Registry (at the time of enrollment) was greater, at 61 years old (Interquartile range, IQR 14), than the participants in PRO-ACT (at the time of recruitment) at 57 years old (IQR 17). Sixty to 64% of the participants in both databases were between 50 and 69 years old. Nonetheless, PRO-ACT had greater representation of younger (≤ 59 years old) participants at about 57% compared to the Registry enrollees (43%). Similarly, when examining the age distribution of the research study participation in the Registry portal database alone, a greater proportion of those less than 50 years of age reported ever participating in ALS research studies at 28.6% (vs. 20.2%) than the older age groups (Table 2).

**Table 1:** Subject demographics in PRO-ACT (data updated August of 2022) and National ALS Registry web portal databases (2010–2021)

		Registry			PRO-ACT			
		*n*	%	95% CI	*n*	%	95% CI	*p*-value
Total		10,471			11,675			
Age (yrs) at enrollment, median (IQR)		61 (14)			57 (17)			< 0.001^a^
Age group (years)	18-39	374	3.6	3.2, 3.9	867	9.0	8.5, 9.6	< 0.001^b^
	40-49	1213	11.6	11.0, 12.2	1786	18.6	17.8, 19.4	
	50-59	2959	28.3	27.4, 29.1	2783	29.0	28.1, 29.9	
	60-69	3718	35.5	34.6, 36.4	2940	30.6	29.7, 31.6	
	70-79	1897	18.1	17.4, 18.9	1167	12.2	11.5, 12.8	
	80+	310	3.0	2.7, 3.3	55	0.6	0.4, 0.8	
Sex	Female	4257	40.7	39.7, 41.6	4579	39.2	38.4, 40.1	0.032^b^
	Male	6214	59.3	58.4, 60.3	7090	60.8	59.9, 61.6	
Ethnicity	Hispanic	332	3.2	2.9, 3.6	200	7.0	6.1, 7.9	< 0.001^b^
	Non-Hispanic	10,016	96.8	96.4, 97.1	2677	93.1	92.1, 93.9	
Race	White	9662	94.2	93.7, 94.6	7578	95.3	94.9, 95.8	< 0.001^b^
	Black/African American	239	2.3	2.1, 2.6	136	1.7	1.5, 2.0	
	Asian	134	1.3	1.1, 1.6	73	0.9	0.7, 1.2	
	American Indian/Alaska Native	43	0.4	0.3, 0.6	9	0.1	0.1, 0.2	
	Hawaiian or Pacific Islander	9	0.1	0.1, 0.2	1	< 0.05	0.0, 0.1	
	Other or Multirace^c^	169	1.7	1.4, 1.9	151	1.6	1.6, 2.2	
Race known		10,256	98.0	97.7, 98.2	7948	68.1	67.2, 68.9	< 0.001^b^
Race unknown^d^		215	2.1	1.8, 2.3	3727	31.9	31.1, 32.8	

^a^Wilcoxon Rank-Sum; ^b^Pearson's chi-square

^c^Other or Multirace: Those who self-reported or having a race record as “Other” or checked more than one race category

^d^Unknown: Observations as recorded as “Unknown” or missing race information

**Table 2:** Percentage of the National ALS Registry (2010–2021) enrollees stratified by age groups with history of participation in ALS research studies^a^

Age group (years)	Ever participated in ALS research studies^a^			
	*n*/total	Percent^b^	95% CI	*p*-value^c^
Younger than 50	153/535	28.60	24.93, 32.58	< 0.001
50 and older	685/3388	20.22	18.90, 21.61	

^a^Type of research studies is not defined—research studies can be clinical trials and/or epidemiological studies

^b^Percent of registrants with a history of ALS research study participation in the corresponding age group

^c^Pearson’s chi-square

Participants in both databases were predominantly male, non-Hispanic, and White. While more participants of Hispanic ethnicity were found in the PRO-ACT database (7.0% vs 3.2%), Black/African American (2.3% vs. 1.7%) and American Indian/Alaska Native (0.4% vs 0.1%) were found in greater percentages in the Registry portal (Table 1). The difference between other race categories was not statistically significant. However, racial information was captured at a different percentage in the studied databases. A statistically significant large number of clinical trial recruits in the PRO-ACT database had missing or unknown race information compared to the Registry database (31.9% vs 2.1%, Table 1). When research study participation was examined among different ethnicity and racial groups in the Registry enrollees, there was a greater, but not significantly different, participation by those of Hispanic ethnicity (25.0% vs. 21.3%) and White (21.6%) compared to other groups (Table 3).

**Table 3:** Percentage of the National ALS Registry (2010–2021) enrollees stratified by ethnicity and race groups with a history of participation in ALS research studies^a^

	Ever participated in ALS research studies^a^			
	Percent	Standard error	95% CI	*p*-value^b^
Ethnicity (total = 3923)				0.399
Hispanic	25.0	4.0	18.0, 33.7	
Non-Hispanic	21.3	0.6	20.0, 22.6	
Race group (total = 3899)				0.232
White	21.6	0.7	20.3, 23.0	
Black/African American	14.5	4.2	8.0, 24.9	
Asian and Others^c^	17.0	3.2	11.6, 24.3	

^a^Type of research studies is not defined—research studies can be clinical trials and/or epidemiological studies

^b^Pearson’s chi-square

^c^Asian and others: those who self-reported as “Asian,” “American Indian/Alaska Native,” “Other,” or checked more than one race category; no observation for Hawaiian or Pacific Islanders

Percentages of participants with lumbar (71.5% vs 72.2%) or bulbar (22.1% vs. 22.5%) onset were similarly reported (Table 4). The median age at symptom onset (60 vs. 55 years old) and at ALS diagnosis (60 vs. 56 years old) was higher in the Registry data compared to PRO-ACT. While time between self-reported symptom onset and diagnosis was longer in Registry participants (11 vs. 9 months), self-reported ALSFRS-R total scores were higher (34 vs. 29). Riluzole use (80.3% vs. 64.7%) and family history of ALS were reported at a greater proportion in the participants of PRO-ACT compared to the Registry (18.3% vs. 6.2%, Table 4).

**Table 4:** Subject symptom onset characteristics in PRO-ACT (data updated August of 2022) and National ALS Registry (2010–2021) web portal databases

		Registry			PRO-ACT			
		*n*	%	95% CI	*n*	%	95% CI	p-value
Site of onset	Limb	2816	71.5	70.1, 72.3	7415	72.2	71.3, 73.1	0.027^b^
	Bulbar	870	22.1	20.8, 23.4	2312	22.5	21.7, 23.3	
	Both or Others^1^	253	6.4	5.7, 7.2	541	5.3	4.9, 5.7	
Age (yrs) at symptom onset, median (IQR)		60 (14)			55 (17)			< 0.001^a^
Age (yrs) at diagnosis, median (IQR)		60 (14)			56 (17)			< 0.001^a^
Time between symptom onset and diagnosis (months), median (IQR)		11 (13)			9.0 (8)			< 0.001^a^
ALSFRS-R, median (IQR)		34 (13)			29 (16)			< 0.001^a^
Riluzole use	Yes	2532	64.7	63.2, 66.2	7614	80.3	79.5, 81.1	< 0.001^b^
	No	1381	35.3	33.8, 36.8	1864	19.7	18.9, 20.5	
Family^2^ history of ALS	Yes	472	6.2	5.7, 6.8	190	18.3	16.1, 20.8	< 0.001^b^
	No	7107	93.8	93.2, 94.3	849	81.7	79.2, 84.0	

^1^Others reported (bulbar and limb: neck, back, stomach, breathing muscle, all over the body)

^2^Family member may include biological mother, father, sister, brother, or children

^a^Wilcoxon rank sum; ^b^Pearson’s chi-square

## Discussion

In April 2022, the FDA published a guidance for the specific purpose of improving racial and ethnic diversity and inclusion from disproportionately under-represented populations in clinical trials [33]. However, it is also recommended to diversify the inclusion of patient characteristics such as age, sex, socioeconomic status, geography, varying physical and cognitive abilities, and co-morbidities as they can provide important information when evaluating the safety and efficacy of the medical device or therapeutics. Whether this FDA guidance will be implemented in the future is pending as of date, but the importance of reinforcing the diverse representation in clinical trials is realized [34].

Clinical characteristics of those enrolled in the Registry between 2010 and 2015 have been previously described [35]. Here, we analyzed the Registry portal data updated through 2021 and compared the demographics and ALS-related characteristics with the study participants compiled in PRO-ACT. We found a statistically significant difference in the age distribution, where a greater proportion of Registry enrollees were older than 60 years of age, while those younger than 50 were more represented in the PRO-ACT database. These age differences are expected since the PRO-ACT database is comprised of clinical trial data, which is inherently skewed to a younger cohort resulting from potentially strict eligibility criteria. Similarly, this pattern is also seen among the Registry enrollees reporting ever participating in the research studies.

Historically, improving representation of elderly adults in clinical trials has been challenging and lacking across all disciplines of clinical trials [30, 32, 36–38]. Older participants are more likely to have co-morbidities, increased risk potential for complications from drug interaction with medications they are currently taking for age-related health conditions, and have other attributes that may confound with outcome assessment [33, 36–39]. Such variables may introduce undesirable reactions to drug response and efficacy measurements which may weaken the robustness of overall data. However, the lack of inclusion of the geriatric participants may demonstrate poor generalizability of the drug effectiveness in the real world, outside of controlled research setting [40, 41].

ALS is a rare disease affecting all genders, races, age groups, and geography. Prevalence and incidence rates of ALS may vary globally [13, 16, 42, 43], owing to differences largely in genetic background, disease surveillance, and case ascertainment methods. Nonetheless, pieces of literature characterize the disease mainly affecting non-Hispanic, White, male, and those between 55–70 years of age. Analyses of U.S. administrative national databases [9–15] and independent analysis of state and metropolitan ALS cases [44] reported similar patterns of demographic attributes.

As expected, over 94% of participants in our analysis were White. The representation of minority groups was small in both databases, but the percentages of Black/African American and American Indian/Alaska Native groups were greater in the Registry. Surprisingly, over 31% of the racial information was either missing or unknown in the PRO-ACT database. A consideration of unknown race in the estimation decreases the White demographic to 65% (data not shown) for the PRO-ACT database. It is unclear how the race information is recorded for those recruited for clinical trials, and what role racial background plays in the clinical trial design or outcome assessment.

The study also evaluated participation by minority group compared to White race group among the Registry enrollees. Black/African Americans self-reported the least participation in ALS research studies compared to other race, although a large margin of error did not yield a significant difference in comparison. We also saw slightly greater percentages in Hispanic than non-Hispanic to ever participate in ALS research studies, though this was not significantly different. Improving the recruitment of under-represented racial groups is one of the key areas addressed in the Federal incentive [33] and continuously identified as a lacking characteristic of clinical trials [31, 34, 45, 46]. Some studies indicate less willingness and a greater presence of fear in African Americans or Hispanics to participate in medical research studies [47–49]. This is compounded by other factors such as social inequity, lack of awareness of trials, mistrust in the healthcare system, and inadequate communication [46, 49]. However, other studies found minorities to be just as willing to participate in medical research [48, 50]. In our comparison among the Registry enrollees, we also found differences in research study participation to be negligible.

In assessing the ALS-associated clinical data, the limb was the most reported site of onset. Lower age at symptom onset and at diagnosis and shorter symptom duration until medical diagnosis reported for PRO-ACT participants reflect strict, or preferred, eligibility criteria of ALS clinical trials [22]. But this distinction too is becoming less relevant when considering the disease heterogeneity of ALS, with different clinical phenotypes and progression rates, and variable responses to medications and treatments [25, 51]. Surprisingly, we saw higher self-reported ALSFRS-R scores in the Registry data than in the PRO-ACT. ALSFRS-R has been evaluated as a good prognostic marker for disease severity and ALS progression [52–55]. But patients perceive ALSFRS-R questions to be difficult in understanding when self-evaluating the functional capabilities [56]. This subjectivity can inaccurately reflect disease severity or potentially introduce unintended bias with the professionally administered evaluation, as would have been likely for the participants of PRO-ACT. Indeed, a study evaluating inter- and intra-rater variability in the self-administration version of ALSFRS-R saw patients reporting a higher score than the evaluators despite acceptable inter- and intra-rater reliability and reproducibility [53]. Furthermore, a potential rater variability in evaluation performance among the trained health professionals may be present which can arise from variable training intervals and mode of training [57].

Riluzole has been a standard of care and treatment for ALS since its approval [5]. According to one retrospective study, the benefit of riluzole was the greatest for those in late-stage disease at a moderate dosage of 100 mg/day [58] as measured by time spent at that specific stage, while a separate study indicated an early intervention was associated with increased survival rate by 2 months [59]. Our assessment showed a greater proportion of riluzole use in PRO-ACT participants compared to the Registry enrollees (80.3% vs 64.7%). This discrepancy may be partially explained by the larger cohort of younger patients in the PRO-ACT database. A study on factors influencing riluzole prescription among ALS patients in a long-term stay home in Ontario has found age to be negatively associated with riluzole use [60]. Another study investigating riluzole prescription among participants from the Clinical Audit Research and Evaluation of Motor Neuron Disease platform has shown older patients not offered riluzole as a treatment option [61]. It is possible that Registry participants use different medications (e.g., edaravone) other than riluzole, or may have discontinued the use, which may explain the lower riluzole use percentage. Unfortunately, a comparison with the PRO-ACT database is not feasible due to a lack of identifying variables for medications other than riluzole in both databases.

About 10% of ALS cases are attributable to an inheritable genetic cause while other cases have undetermined or little-known genetic and non-genetic etiology. A mutation in *C9ORF72* is the most common genetic trait of familial ALS patients of Caucasian/European background [62, 63] compared with other genes such as *SOD1*, *TARDBP*, and *FUS* to a lesser prevalence [64]. But the magnitude of a familial genetic link differs based on the region and ethnic/racial background of the sample population being studied [62, 64, 65]. Family history of ALS was reported in greater percentage among the recruits of PRO-ACT at 18.3% compared to 6.2% for the Registry. With genetic screening available to detect if family members of ALS patients carry the mutation, gene therapy clinical trials often target the pre-symptomatic carriers of the pathogenic hereditary mutation form [66]. While those clinical trials may have a specific experimental design and target such a cohort, at least one other study has noted patients with familial ALS history are often excluded from the trials [22]. It is unknown how many of the clinical trials represented in PRO-ACT databases have specifically recruited those with a family history of ALS, as with other specifics and the eligibility criteria for the studies.

## Limitations

While the PRO-ACT database represents a single source of publicly available de-identified, merged multi-year records of ALS patients participating in clinical trials, the data are from a limited number of consortia. The database is not inclusive of participants of all available ALS clinical trials that have been conducted in the past or currently being conducted. There is an inherent variability introduced in the effort to harmonize trial data consolidated under different research study protocols. Despite a large sample size, different data collection methods and data standardization may have introduced variance overall.

Unlike cancer and many communicable diseases [67], ALS is not considered a notifiable disease in the U.S. This makes capturing ALS cases at the local, state, and national level challenging, which becomes a significant barrier to ascertaining a complete case count for the estimation of disease burden in the U.S. In addition, our recent evaluation of ALS cases among the U.S. adult population using capture-recapture methods revealed missing case ascertainment of about 27% [15]. Those underrepresented in the Registry may be younger ALS patients and those who have health insurance through private sectors. They are less likely to be captured by the national administrative database such as CMS, VHA, or VBA. Data in the Registry portal are self-reported while data of patients recruited in clinical trials in the PRO-ACT database may have been recorded or determined by the medical professionals from clinical examinations during trial visits. In addition, the portal data consist of information collected in 2010–2021 while the PRO-ACT database consists of clinical trial data donated by participating consortia since 1990 with the latest data publicly made available in August 2022. The latest year of collection is not provided. While these two databases are not mutually exclusive, it is not feasible to verify the presence of a same individual in both databases as personal identification is not disclosed for deduplication.

## Conclusion

Addressing the discrepancy of the demographic niche in both the general ALS patient population as recognized in the Registry portal and those being recruited for clinical trials may increase the generalizability of study outcomes. Considering the shortcoming of racial diversity and inclusion in clinical trials for therapeutics in other disciplines, our findings in participant characteristics reflect a similar pattern of concerns. The analysis has shown an underrepresentation of elderly patients and minority populations in clinical trials and incomplete racial background information. In addition, there was a disproportionate subgroup of different disease duration and progression. Clinical trial enrollment may be limited by preset criteria. Nonetheless, refinement of study patient preference may be needed to allow for greater participation of the general ALS population, as those excluded from the clinical trial enrollment represent a patient population with unmet needs. The Registry portal provides a tool to improve the recruitment effort targeting patients that are often understudied. Such an improvement could then enhance the ability of researchers to diversify the patient pool for clinical trials.

## Supplementary Information

Below is the link to the electronic supplementary material.

Supplementary file1 (DOCX 14.3 KB)

## Data Availability

Data for PRO-ACT is publicly available at https://ncri1.partners.org/ProACT/Home/Index upon approval of request. De-identified data of participants of the National ALS Registry web portal, including the data used for current study, are available upon reasonable request through an application process at https://www.cdc.gov/alsresearch/ResearchersandClinicians_ResearcherRequests.html.
