# Correction to: Comparison of Demographics: National Amyotrophic Lateral Sclerosis Registry and Clinical Trials Data

**DOI:** 10.1007/s40615-024-02110-0

**Published:** 2024-07-30

**Authors:** Moon Han, Jaime Raymond, Theodore C. Larson, Paul Mehta, D. Kevin Horton

**Affiliations:** Office of Innovation and Analytics, Agency for Toxic Substances and Disease Registry/Centers for Disease Control and Prevention, 4770 Buford Hwy NE, Atlanta, GA 30341 USA

**Correction to: Journal of Racial and Ethnic Health Disparities**

10.1007/s40615-024-02047-4

In Table 3 in the article as originally published, the value for the Race group total was incorrectly reported. The correct total is 3899 instead of 3.899.

The original article has been corrected.

