# Supplementary material for: Comparison of Demographics: National Amyotrophic Lateral Sclerosis Registry and Clinical Trials Data
Source: J Racial Ethn Health Disparities. 2024 Jul 8;12(4):2270–8. doi: 10.1007/s40615-024-02047-4 (PMC12241206; doi:10.1007/s40615-024-02047-4)
Supplement: Supplementary file 1 — Supplementary file1 (DOCX 14.3 KB) [file 40615_2024_2047_MOESM1_ESM.docx]

Supplemental Methods

# Comparison of Demographics: National Amyotrophic Lateral Sclerosis Registry and Clinical Trials Data

Moon Han, PhD, MPH, MS1, Jaime Raymond, MPH1, Theodore C. Larson, MS1, Paul Mehta, MD1, D. Kevin Horton, DrPH, MSPH1

1Office of Innovation and Analytics, Agency for Toxic Substances and Disease Registry/Centers for Disease Control and Prevention, Atlanta, GA

Corresponding author:

Moon Han, PhD, MPH, MS Email: [ptr6@cdc.gov](mailto:ptr6@cdc.gov)

Address: 4770 Buford Hwy NE, Atlanta, GA 30341

# Study variables

For the race variable, White or Caucasian was categorized as “White”, Black/African American as “Black/African American”, Asian and its subgroups (e.g., Chinese, Asian Indian, Korean, Filipino, Vietnamese, Other Asian) as “Asian”, and other minority races reported as “American Indian (or Native American)/Alaska Native” and “Hawaiian or Pacific Islander” were reported as its own category. Races recorded or self-reported as others or anyone who reported more than one race were categorized as “Others or Multiracial”.

Symptom onset sites not labeled as “bulbar”, “limb”, “speech and/or swallowing muscles”, or “arm or hand, and leg or foot” were coded as others. Family history of ALS was coded for any immediate family member (mother, father, sister, brother, child) with an ALS diagnosis, whether self-reported (the Registry portal database) or recorded in clinical history (the PRO-ACT database). Symptom duration was calculated from the time of symptom onset to the time of diagnosis in months.
